# Spectroscopic Studies of Copper, Silver and Gold-Metallothioneins

**DOI:** 10.1155/MBD.1994.375

**Published:** 1994

**Authors:** Martin J. Stillman, Anthony Presta, Ziqi Gui, De-Tong Jiang

**Affiliations:** 1 Department of Chemistry University of Western Ontario London Ontario N6A 5B7 Canada

## Abstract

Metallothionein is a ubiquitous protein with a wide range of proposed physiological roles, including the transport, storage and detoxification of essential and nonessential trace metals. The amino acid sequence of isoform 2a of rabbit liver metallothionein, the isoform used in our spectroscopic studies, includes 20 cysteinyl groups out of 62 amino acids. Metallothioneins in general represent an impressive chelating agent for a wide range of metals. Structural studies carried out by a number of research groups (using ^1^H and ^113^Cd NMR, X-ray crystallography, more recently EXAFS, as well as optical spectroscopy) have established that there are three structural motifs for metal binding to mammalian metallothioneins. These three structures are defined by metal to protein stoichiometric ratios, which we believe specifically determine the coordination geometry adopted by the metal in the metal binding site at that metal to protein molar ratio. Tetrahedral geometry is associated with the thiolate coordination of the metals in the M_7_-MT species, for M = Zn(II), Cd(II), and possibly also Hg(II), trigonal coordination is proposed in the M_11-12_-MT species, for M = Ag(I), Cu(I), and possibly also Hg(II), and digonal coordination is proposed for the metal in the M_17-18_-MT species for M = Hg(II), and Ag(I). The M_7_-MT species has been completely characterized for M = Cd(II) and Zn(II). ^113^Cd NMR spectroscopic and x-ray crystallographic data show that mammalian Cd_7_-MT and Zn_7_-MT have a two domain structure, with metal-thiolate clusters of the form M_4_(S_cys_)_11_ (the α domain) and M_3_(S_cys_)_9_ (the β domain). A similar two domain structure involving Cu_6_(S_cys_)_11_ (α) and Cu_6_(S_cys_)_9_ (β) copper-thiolate clusters has been proposed for the Cu_12_-MT species. Copper-, silver- and gold-containing metallothioneins luminesce in the 500-600 nm region from excited triplet, metal-based states that are populated by absorption into the 260-300 nm region of the metal-thiolate charge transfer states. The luminescence spectrum provides a very sensitive probe of the metal-thiolate cluster structures that form when Ag(I), Au(I), and Cu(I) are added to metallothionein. CD spectroscopy has been used in our laboratory to probe the formation of species that exhibit well-defined three-dimensional structures. Saturation of the optical signals during titrations of MT with Cu(I) or Ag(I) clearly show formation of unique metal-thiolate structures at specific metal:protein ratios. However, we have proposed that these M=7, 12 and 18 structures form within a continuum of stoichiometries. Compounds prepared at these specific molar ratios have been examined by X-ray Absorption Spectroscopy (XAS) and bond lengths have been determined for the metal-thiolate clusters through the EXAFS technique. The stoichiometric ratio data from the optical experiments and the bond lengths from the XAS experiments are used to propose structures for the metal-thiolate binding site with reference to known inorganic metal-thiolate compounds.


### SPECTROSCOPIC STUDIES OF COPPER, SILVER AND GOLD-METALLOTHIONEINS

Martin J. Stillman*, Anthony Presta, Ziqi Gui, and De-Tong Jiang

Department of Chemistry, University of Western Ontario, London, Ontario, N6A 5B7 Canada

### *ABSTRACT*

Metallothionein is a ubiquitous protein with a wide range of proposed physiological roles, including the transport, storage and detoxification of essential and nonessential trace metals. The amino acid sequence of isoform 2a of rabbit liver metallothionein, the isoform used in our spectroscopic studies, includes 20 cysteinyl groups out of 62 amino acids. Metallothioneins in general represent an impressive chelating agent for a wide range of metals. Structural studies carried out by a number of research groups (using $^1$H and $^{113}$Cd NMR, X-ray crystallography, more recently EXAFS, as well as optical spectroscopy) have established that there are three structural motifs for metal binding to mammalian metallothioneins. These three structures are defined by metal to protein stoichiometric ratios, which we believe specifically determine the coordination geometry adopted by the metal in the metal binding site at that metal to protein molar ratio. Tetrahedral geometry is associated with the thiolate coordination of the metals in the $M_7$-MT species, for M = Zn(II), Cd(II), and possibly also Hg(II), trigonal coordination is proposed in the $M_{11-12}$-MT species, for M = Ag(I), Cu(I), and possibly also Hg(II), and digonal coordination is proposed for the metal in the $M_{17-18}$-MT species for M = Hg(II), and Ag(I). The $M_7$-MT species has been completely characterized for M = Cd(II) and Zn(II). $^{113}$Cd NMR spectroscopic and x-ray crystallographic data show that mammalian $Cd_7$-MT and $Zn_7$-MT have a two domain structure, with metal-thiolate clusters of the form $M_4(S_{cys})_{11}$ (the $\alpha$ domain) and $M_3(S_{cys})_9$ (the $\beta$ domain). A similar two domain structure involving $Cu_6(S_{cys})_{11}$ ($\alpha$) and $Cu_6(S_{cys})_9$ ($\beta$) copper-thiolate clusters has been proposed for the $Cu_{12}$-MT species. Copper-, silver- and gold-containing metallothioneins luminesce in the 500-600 nm region from excited triplet, metal-based states that are populated by absorption into the 260-300 nm region of the metal-thiolate charge transfer states. The luminescence spectrum provides a very sensitive probe of the metal-thiolate cluster structures that form when Ag(I), Au(I), and Cu(I) are added to metallothionein. CD spectroscopy has been used in our laboratory to probe the formation of species that exhibit well-defined three-dimensional structures. Saturation of the optical signals during titrations of MT with Cu(I) or Ag(I) clearly show formation of unique metal-thiolate structures at specific metal:protein ratios. However, we have proposed that these M=7, 12 and 18 structures form within a continuum of stoichiometries. Compounds prepared at these specific molar ratios have been examined by X-ray Absorption Spectroscopy (XAS) and bond lengths have been determined for the metal-thiolate clusters through the EXAFS technique. The stoichiometric ratio data from the optical experiments and the bond lengths from the XAS experiments are used to propose structures for the metal-thiolate binding site with reference to known inorganic metal-thiolate compounds.

### *Introduction*

Studies of the rich biochemistry and chemistry of metallothionein began following the initial discovery and characterization of a cadmium and zinc binding protein from horse kidney by Margoshes and Vallee in 1957 [1]. This protein was called metallothionein by Kagi and Vallee in 1960 and the first spectral properties were reported in 1961 [2,3]. Since that time, there has been a rapid and extensive study of all aspects of the biochemical and chemical properties of metallothioneins isolated from a wide variety of species. A series of monographs and proceedings have appeared at timely intervals that provide a great many details and trace the development of our knowledge about the metallothioneins [4].

Metallothioneins have been isolated from vertebrates, invertebrates and microorganisms, and are classified in terms of three classes [4]. In each case the protein is identified by a very high cysteine content (30%), low molecular weight (3,000 - 10,000 daltons), absence of aromatic amino acids, the presence of several cys-x-cys sequences in the primary structure and, of greatest significance, the property of binding metals in metal-thiolate-cluster structures that involve 3, 4, or 6 metals. Class I metallothioneins include about 60 proteins isolated from vertebrates and crustaceans, each of which having a primary peptide sequence with 60 to 62 amino acid residues. The class I mammalian proteins are generally characterized by formation of two metal binding domains, named α and β, with zinc, cadmium and copper [4]. The class II peptides are isolated from yeasts, fungus, and plants, while class III metallothioneins are characterized as γ-glutamyl isopeptides or phytochelatins. The structure of the metal binding site in mammalian metallothioneins was determined initially by Otvos and Armitage using $^{113}Cd$ NMR techniques on $Cd_7$-MT isolated from rabbit livers [5]. Later, both $^1H$ NMR [6] and x-ray crystallographic [7] techniques established the connectivities between the cysteinyl thiolates, $S_{cys}$, and the seven cadmium and zinc atoms. Figures 1 and 2 shows the sequence and metal-$S_{cys}$ connectivities for $Cd_7$-MT 2a based on data of Kagi [8]. Figure 2 shows a representation of the structure for $M_7$-MT, where M=Cd(II) and Zn(II), for rabbit liver MT 2a, a 62 amino acid peptide, based on the $^{113}Cd$ NMR, $^1H$ NMR, and x-ray results [4].

In the mammalian protein, 8 of the 20 $S_{cys}$ form bridging bonds with two metals, while 12 $S_{cys}$ form terminal bonds. The $M_7$-MT structures in the mammalian protein comprise two metal binding domains, $M_3(S_{cys})_9$ named the β domain, and $M_4(S_{cys})_{11}$ named the α domain [5,7,9], With the peptide chain wrapped round the outside of the binding sites, we see that the $S_{cys}$-M-$S_{cys}$ bonds act to crosslink the peptide chain forming two, three-dimensional cores for the metal binding sites in the metallated protein. The metals bound to mammalian metallothionein can be exchanged by addition of a metal with a greater binding constant. The observed order of binding strengths for mammalian metallothioneins is: Zn<Cd<Cu<Hg. In Figure 3, we show the CD spectral patterns recorded as $Cd_7$-MT is formed by direct addition of Cd(II) to aqueous solutions of $Zn_7$-MT. A different spectral pattern is observed if Cd(II) is added to the metal free protein, apoMT. The CD spectral data in Figure 3 show that the Cd(II) displaces the Zn(II) in both domains initially, before saturating with a stoichiometry of 7. Cd(II) adds to apoMT in a domain specific fashion, first into the α domain up to a stoichiometric ratio of 4, then into the β domain up to Cd:MT=7 [10]. These two sets of CD spectral data introduce the two reaction pathways that dominate metal binding in mammalian metallothionein. An incoming metal can bind to either domain preferentially, which is a domain specific pathway, or to both domains on a statistical basis, which is a distributed pathway. Work by Winge et al. [11] has shown that equilibrated metallothionein in which 2 metals are bound exhibits domain specificity. For example, in Cd,Zn-MT, the Cd(II) will be located primarily in the α domain, while Zn(II) will be located primarily in the β domain; in contrast, in Cu,Zn-MT, the Cu(I) will be located in the β domain while the Zn(II) will be located in the α domain. Spectroscopic studies from our laboratory [12] suggest that initial binding is kinetically controlled and the incoming metals bind first in a distributed manner, after time, and at temperatures above about 15 $^0C$, the metals redistribute to form the thermodynamically stable, domain specific product. The $M_7$-MT structure has also been proposed for $Co(II)_7$-MT based on absorption, magnetic circular dichroism (MCD), and electron paramagnetic resonance (EPR) [13].

A second structural motif is found for Ag(I) and Cu(I), in which a metal:MT stoichiometry of 12 has been determined [12b,12c,14]. Typically, in the Cu(I)-containing mammalian metallothioneins it has been proposed from results of a number of spectroscopic techniques [12c,15] that the Cu(I) is trigonally coordinated by the $S_{cys}$, binding as $M_6(S_{cys})_9$ (β domain) and $M_6(S_{cys})_{11}$ (α domain).

Finally, a third motif has been proposed based on results of spectroscopic studies of Hg(II) and Ag(I) binding to rabbit liver metallothionein. $Hg_{18}$-MT 2 is characterized by a unique CD spectrum [16], a coordination number of 2(0.8), with an average Hg-S bond length of 242(3) pm [17]. Similar CD spectral properties have been found for Ag-MT, and $Ag_{17}$-MT 1 has been formed following addition of Ag(I) to rabbit liver metallothionein [18].

The insulation of the two metal-thiolate cluster structures from the solvent results in emission intensity being measured for Cu-MT at room temperature [12b,14b,19] and Ag-MT and Au-MT at cryogenic temperatures [19]. This wide applicability, and dependence on the metal to protein molar ratio, means that emission spectroscopy is a powerful technique with which to study the metal binding chemistry of the Ag(I), Au(I) and Cu(I)-containing proteins both *in vitro* and *in vivo* [20].

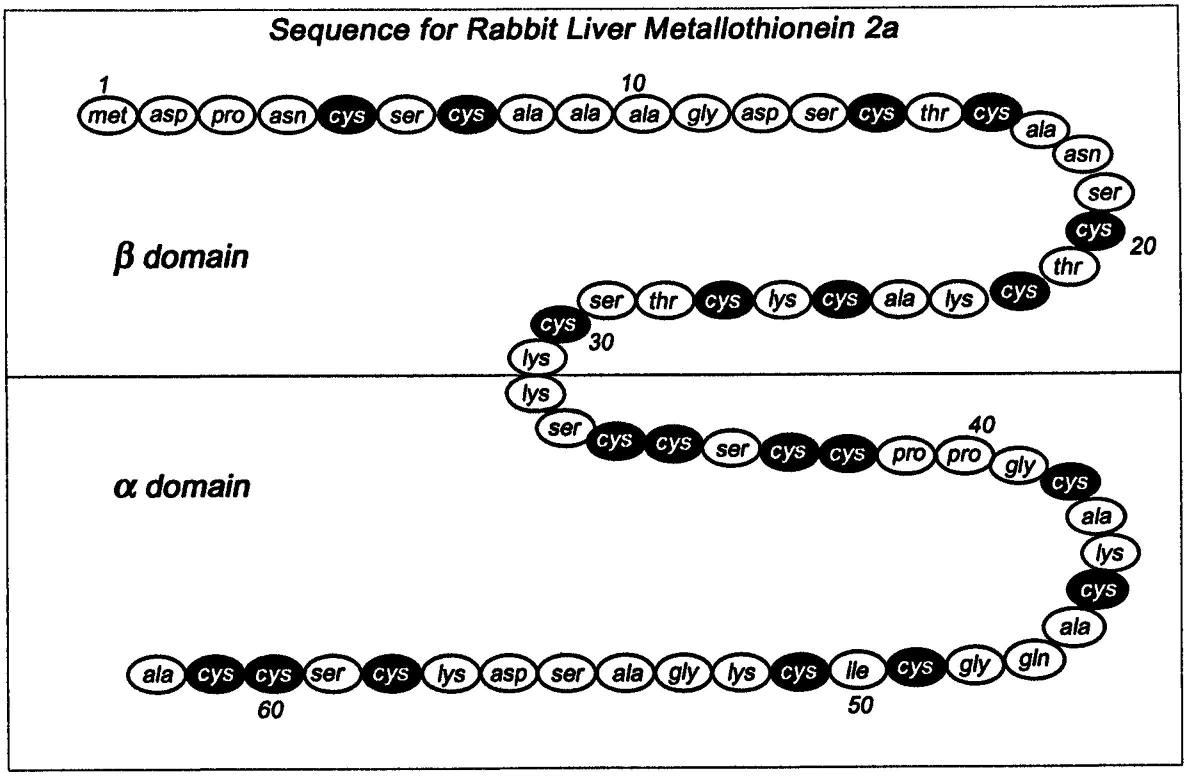

Figure 1. Sequence of rabbit liver metallothionein isoform 2a, based on the data of Kagi [8].

This present paper concerns the spectroscopic characterization of metal binding to rabbit liver metallothionein. Using optical techniques, we can determine with high precision, the metal to protein stoichiometric ratios that result in the formation of distinct, three-dimensionally organized structures within the metal binding site.

### Materials and Methods

For most of the experiments described here, $Zn_7$-MT was isolated from rabbit livers following *in vivo* induction procedures using aqueous zinc salts. In some instances the data were recorded from rat liver protein. The preparative methods were described in the original papers that are cited with the spectral data. Protein was purified using gel filtration and gel electrophoresis techniques [21]. Aqueous protein solutions were prepared by dissolving the protein in argon-saturated distilled water. Protein concentrations were estimated from measurements of the -SH group and zinc concentrations as described previously [22]. These estimations were based on the assumption that there are 20 -SH groups and 7 Zn atoms in each protein molecule. The concentration of -SH groups was determined by the spectrophotometric measurement of the colored thionitrobenzoate anion ($\varepsilon_{420}$ = 13 600 $M^{-1}$ $cm^{-1}$) produced by reaction with DTNB (5,5'-dithiobis(-2-nitrobenzoic acid)) in the presence of 6M guanidine hydrochloride [23]. Zinc concentrations were determined by flame atomic absorption spectroscopy (AAS) using a Varian AA875 atomic absorption spectrophotometer. Very complete isolation and purification details for a range of different metallothioneins have been published as part of the '*Methods in Enzymology*' series [4d].

All the metal titration experiments of Zn-MT were carried out in the same manner. Taking as an example titrations of $Zn_7$-MT with Cu(I), 10 μM solutions of aqueous $Zn_7$-MT were titrated with Cu(I) in the form of $[Cu(CH_3CN)_4]ClO_4$. This salt was prepared by the method of Hemmerich and Sigwart [24] and was dissolved in a 30% (v/v) acetonitrile/water solution. Protein solutions were bubbled with Ar for 5 minutes after each addition of Cu(I) before measuring the spectral data. There is no evidence that the displaced metal ions affect the optical spectra recorded during the titrations. After titrating the protein solution with approximately 20 Cu(I) equivalents, the final $CH_3CN$ concentration was 1 to 1.5 % (v/v). Titrations involving apoMT use multiple solutions, for example, one solution for each Cu(I):MT molar ratio shown.

Circular dichroism spectra were recorded on a Jasco J-500C spectropolarimeter controlled by an IBM S9001 computer using the program CDSCAN [25]. Emission spectra were measured on a Photon Technology Inc. LS-100 spectrometer. Optical glass filters were placed over the excitation (Corning 7-54 or Schott BG-24) and emission slits (Corning CS 3-74 or Schott GG-420) for observation of emission in the 500-700 nm region with excitation at 300 nm.

### *Results and Discussion*

In this paper we will describe CD and emission spectral data that depend entirely on the formation of the metal-thiolate clustered binding site of the 62 peptide metallothionein isolated from rabbit liver. The spectral intensity is observed to depend directly, but often in a nonlinear manner, on the metal to protein molar ratios. The CD experiment provides information about the winding of the peptide chain around the binding site to allow each cysteinyl sulfur to connect to one of the metals. Because the peptide chain wraps round the clustered metal-thiolate structure the possibility exists that the whole structure will be chiral. Unusually for a protein, there are no amino acids that absorb light with wavelengths greater than 230 nm. ApoMT is totally colorless above 230 nm in the absence of metals. Although metals like Cd(II), Zn(II), Ag(I), Au(I), and Cu(I) exhibit no optical transitions within the normal UV-visible wavelength range, the presence of the thiolate ligand results in ligand to metal charge transfer (LMCT). These LMCT transitions lie in the region between 230 and 400 nm. When any transition takes place within a chiral environment, the chirality is imparted on that transition. In particular, there will be intensity enhancement for LMCT transitions that terminate on degenerate states, because under these conditions, exciton coupling can occur. The exciton splitting results in the measurement of a highly characteristic degenerate

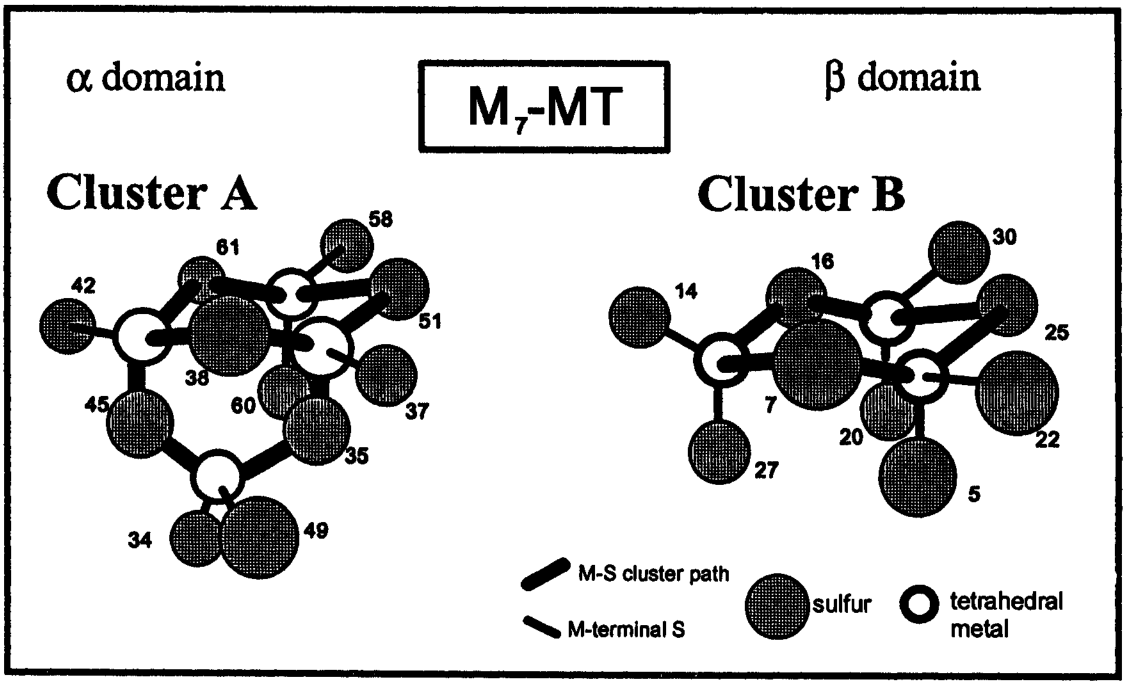

Figure 2. Representation of the three-dimensional structure of the two metal-binding domains in rabbit liver $Cd_7$-MT 2a, based on the data of Otvos and Armitage [5], Messerle et al. [6c], and Robbins et al. [7b].

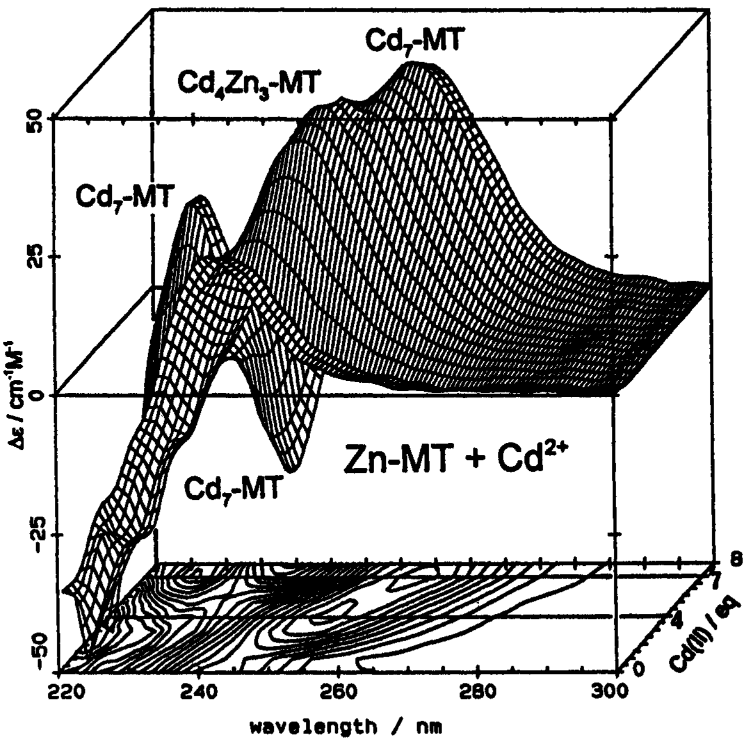

Figure 3. CD spectra recorded as Cd(II) is added to a single solution of rabbit liver $Zn_7$-MT 2 at 23 °C. The CD intensity profile is plotted as a function of the Cd:MT molar ratio and shows that there is a change in spectral characteristics at the Cd:MT=4 point; saturation of the signal occurs at Cd:MT=7. The derivative signal characteristic of $Cd_4$-α, [10] is observed only past Cd:MT=5. Reproduced with permission from Presta et al. [12c].

like band centered approximately on the absorption band maximum. When metals bind either directly to the metal-free, or apoMT, or when an added metal displaces an existing metal, for example when Cu(I) is added to $Zn_7$-MT, the new metal-thiolate cluster structures that form may introduce new chirality or adopt the same chirality as for the initially bound metal. What is important, is how the parameters that control the observed chiral spectrum change as the new metal is bound to the two domains (in mammalian metallothionein). First, both the absorption and CD spectra will exhibit band maxima at the same energy (as the transitions are the same in both techniques), but the CD intensity mechanism is not as sensitive to transitions that do not originate within the chiral structure, so the CD spectrum becomes more sensitive to metal-based transitions for metals *located within the chiral binding site*. Second, because the selection rules for the CD bands involve the magnetic dipole operator as well as the electric dipole operator, the relative intensities of the CD bands may be quite different when compared with the associated absorption spectrum. Third, the sign and magnitude of the individual CD bands will change as the three-dimensional structure of the metal binding site changes. Therefore, in experiments in which a tighter binding metal is added to Zn-MT (for example Cd(II) or Cu(I)), the changes in the CD spectral intensity at every point during the titration directly indicate (i) whether the binding site structure is maintained as with the Zn(II) (the CD envelope will remain the same but shifted to a different wavelength), (ii) whether a new structure forms under the influence of the incoming metal (a new CD envelope will be observed, also at a different energy), or (iii) whether the binding site opens up and the three-dimensional structure is lost (the CD signal will be essentially quenched as a function of the molar ratio of the incoming metal, or the addition of a competitive ligand).

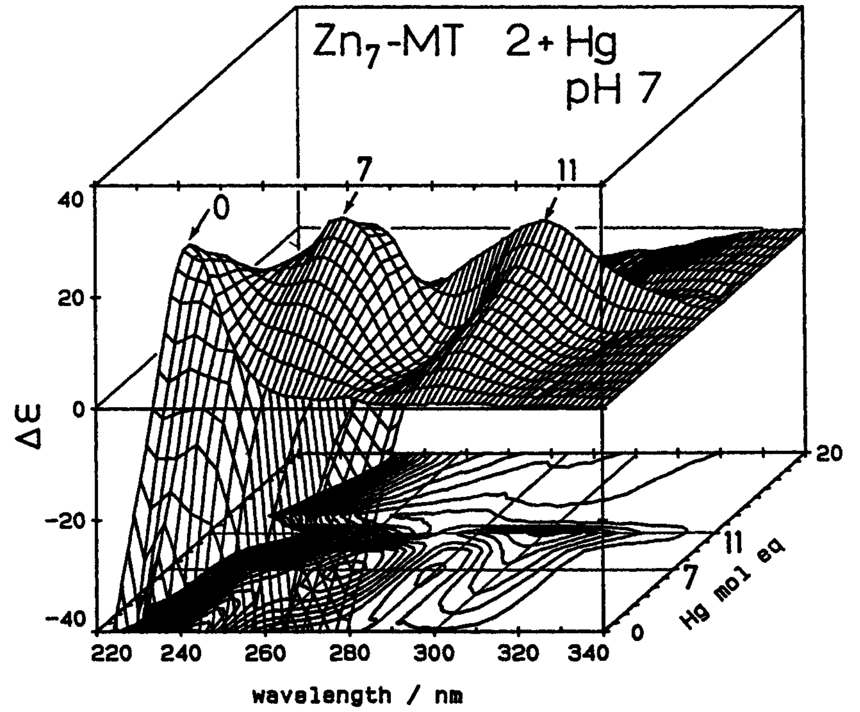

Figure 4. CD spectral profile plotted as a function of the Hg:MT molar ratio as Hg(II) was added to a single solution of rabbit liver $Zn_7$-MT 2 at pH 7 and room temperature. The profile shows the development of the $Hg_7$-MT and $Hg_{11}$-MT species. Reproduced with permission from Lu and Stillman [27].

We can now summarize the key features of the CD experiment as used in probing the formation of ordered metal-thiolate structures in the binding site: the CD spectrum depends on the chirality of the metal binding site as a whole, the spectrum to the red of 230 nm is entirely dependent on the bound metals, and there is no contribution from metal ions in solution (but which might contribute to the absorbance measured).

Figure 3 illustrates the very great sensitivity of the CD spectrum to changes that take place in the metal binding site. In a titration of $Zn_7$-MT with Cd(II), the Cd(II) first binds to both domains leading to mixed, Cd,Zn-thiolate clusters [10,12c]. The dramatic spectral changes observed at the Cd:MT = 5 point indicate that complete $Cd_4S_{11}$ groups begin to form in the $\alpha$ domain at this stage. Warming the solution with only 4 Cd(II) added will generate the domain specific product as Cd(II) in the $\beta$ domain migrates to the $\alpha$ domain [12a].

From the CD spectroscopic data we obtain precise data on the metal to protein stoichiometric ratio. These data indicate that for Ag(I), Cu(I), and Hg(II), a number of different complexes form during the titration. Saturation points in the spectral intensities indicate that a preferred structure has formed. However, with metals like Ag(I) and Cu(I), changes in coordination number can result in a new coordination chemistry being observed as the metal to protein molar ratio increases. The clearest indication that a metal bound in the binding site of metallothionein might exhibit more than one coordination number successively in a titration is seen in the titrations of $Zn_7$-MT and apoMT with Hg(II). In Figures 4 and 5, the CD intensity profile is plotted as a function of the Hg:MT molar ratio, first when Hg(II) is added to Zn-MT 2 at pH 7. Quite complicated speciation is observed in the set of CD spectra when the intensity profile is plotted as a function of the Hg:MT molar ratio. First, the bands due to Zn-S near 240 nm, are replaced by bands due to Hg-S at 260 and 300 nm. However, the structure that forms at Hg:MT=7 is not as well defined as when Hg(II)

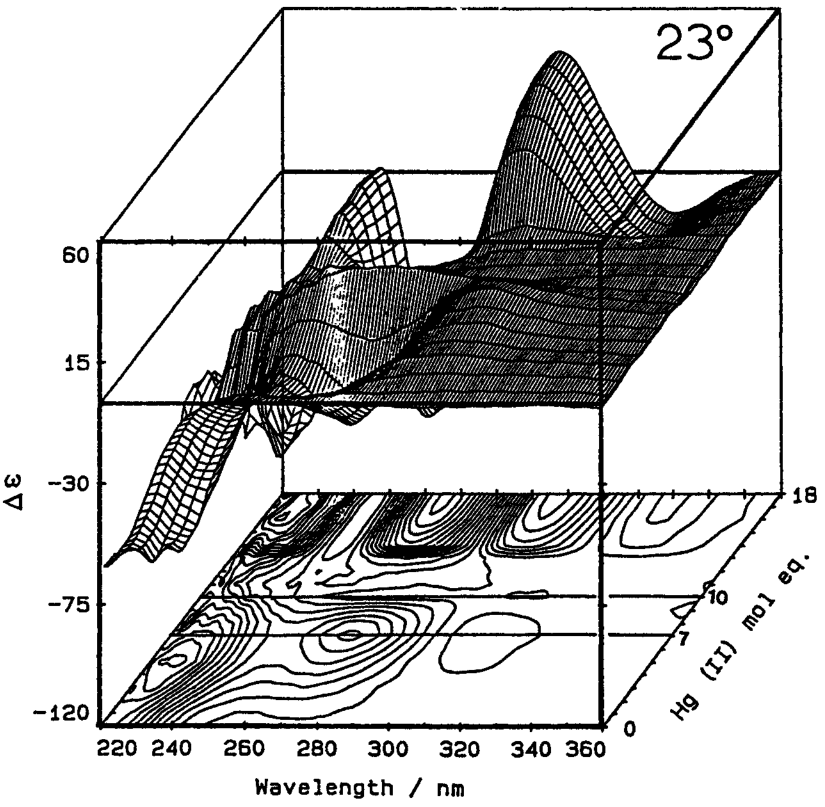

Figure 5. CD spectral profile plotted as a function of the Hg:MT molar ratio when Hg(II) binds to rabbit liver apoMT 2 at pH 2. The profile shows the development of the $Hg_7$-MT and $Hg_{18}$-MT species. Reproduced with permission from Lu, et al. [16b].

is added to the apoMT. With Zn-MT, a new species forms as the Hg:MT ratio increases past 7 up to a maximum at 11. The steep collapse in CD intensity at 11 indicates that the domains must unwind at this point under the stimulus of the 12th mercury atom; the CD spectral intensity is lost because the three-dimensional structure breaks up. However, the Hg(II) still binds to the 20 Scys up to Hg:MT=20. Figure 5 shows that a third structure can form but only at pH<7. Now, following saturation in the CD signal at exactly 7, a new species forms at Hg:MT=18. XANES [26] and EXAFS [17] studies indicate that this is a unique structure, possibly the $Hg_{18}S_{20}$ cluster adopts a single domain rather than the two domains anticipated for $Hg_7$-MT and $Hg_{11}$-MT [16b,27].

### Copper Binding to $Zn_7$-MT

Metallothionein binds copper only in the +1 oxidation state [29]. Cu(II) reacts with metallothionein to form Cu(I) and probably denatures the protein through oxidation of the cysteinyl thiolates. In this present study, aliquots of the Cu(I) salt $[Cu(CH_3CN)_4]ClO_4$ were added to aqueous $Zn_7$-MT 2 to replace the tetrahedrally-bound Zn(II) between 3 and 52 °C. These experiments examine the differences in the binding of Cu(I) to metallothionein under kinetic versus thermodynamic conditions. The CD spectra recorded during $Cu^+$ addition to $Zn_7$-MT 2 at the temperatures stated above are shown in Figures 6 and 7, respectively. There are significant differences in the development of the respective spectral envelopes at the different temperatures, indicating a structural change in Cu(I)-binding under the two conditions.

At the low temperature, that is under kinetic control, the CD signals saturate with formation of the $Cu_{12}$-MT and $Cu_{15}$-MT species, Figure 6. At the higher temperature (52 °C), Figure 7, $Cu_9Zn_2$-MT exhibits a unique spectrum, in addition to those for the $Cu_{12}$-MT and $Cu_{15}$-MT species. Raising the temperature from 3 to 52 °C has the effect of resolving the unique structure of the $Cu_9Zn_2$-MT species before forming the Cu(I)-thiolate structures found in $Cu_{12}$-MT and $Cu_{15}$-MT [12c]. The use of CD spectroscopy as a probe during the titration of $Zn_7$-MT with Cu(I) not only signals the formation of a series of well-defined Cu(I)-thiolate structures during Cu(I) addition, but also indicates how the polypeptide changes as Cu(I) replaces the Zn(II). These data show that at low temperatures the polypeptide is constrained in its ability to reorient itself to accommodate trigonally coordinated Cu(I) in place of the tetrahedral Zn(II), thus forming the kinetic product. At higher temperatures, the polypeptide is clearly able to reorient itself at lower Cu(I):MT ratios, allowing the thermodynamic $Cu_9Zn_2$-MT product to be resolved as a distinct structure.

Emission from the copper-containing protein isolated from *Neurospora crassa* was first reported by Beltramini and Lerch [28]. Since then, emission spectra from a wide range of copper, silver, gold and platinum metallothioneins have been described [18-20,29]. Emission spectra from copper metallothioneins from a variety of sources have been studied in great detail. The spectral data shown in Figure 8 illustrate the strong dependence of the emission intensity on the Cu(I) to MT stoichiometry. Because the intensities are plotted in Figure 8 in terms of the intensity per Cu(I) added, the data clearly show that the Cu(I)-thiolate cluster structure becomes dramatically more emissive up to the Cu(I):MT=12 point, then the intensity collapses up to Cu(I):MT=20. We have interpreted this effect in terms of a reduction in solvent quenching as the binding site requires a more tightly wound peptide with increasing metal, which acts to insulate the Cu(I)-thiolate clusters from the solvent. The spectral profiles shown in Figure 8 were recorded at temperature extremes

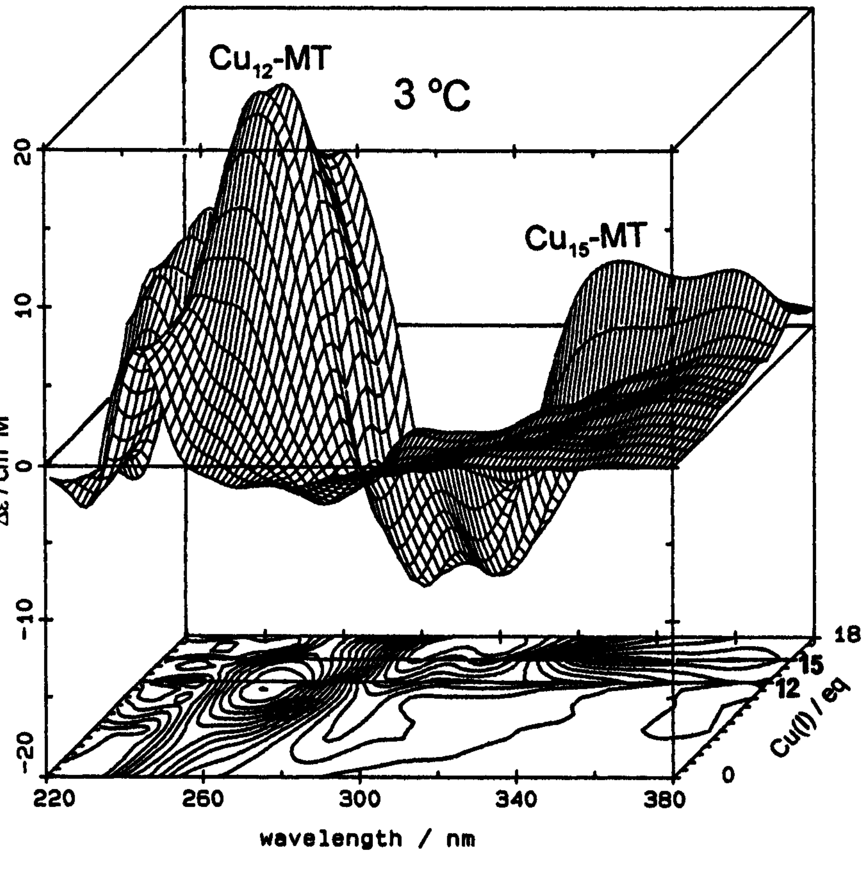

Figure 6. CD spectra recorded as Cu(I) is added to a single solution of rabbit liver $Zn_7$-MT at 3 °C. The CD intensity profile is plotted as a function of the Cu:MT molar ratio and shows that there is a change in spectral characteristics at the Cu:MT= 12 and 15 points; the CD signal intensity collapses at Cu:MT>15. Reproduced with permission from Presta et al., [12c ].

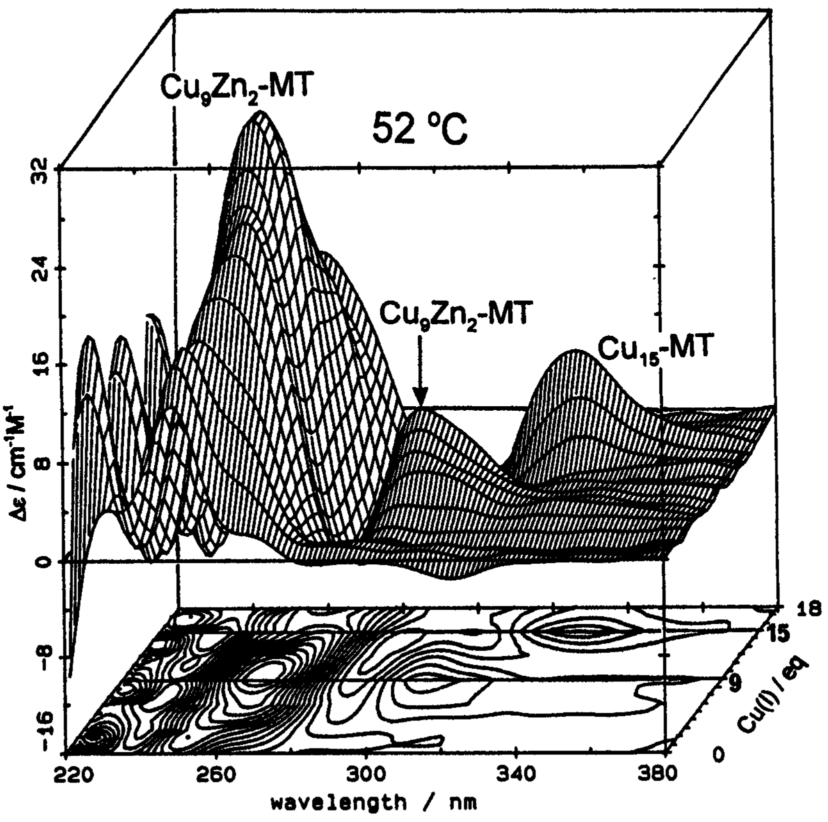

Figure 7. CD spectra recorded as Cu(I) is added to a single solution of rabbit liver $Zn_7$-MT at 52 °C. The CD intensity profile is plotted as a function of the Cu:MT molar ratio and shows that there is a change in spectral characteristics at the Cu:MT= 9 and 15 points; the CD signal intensity collapses at Cu:MT>15. Reproduced with permission from Presta et al., [12c ].

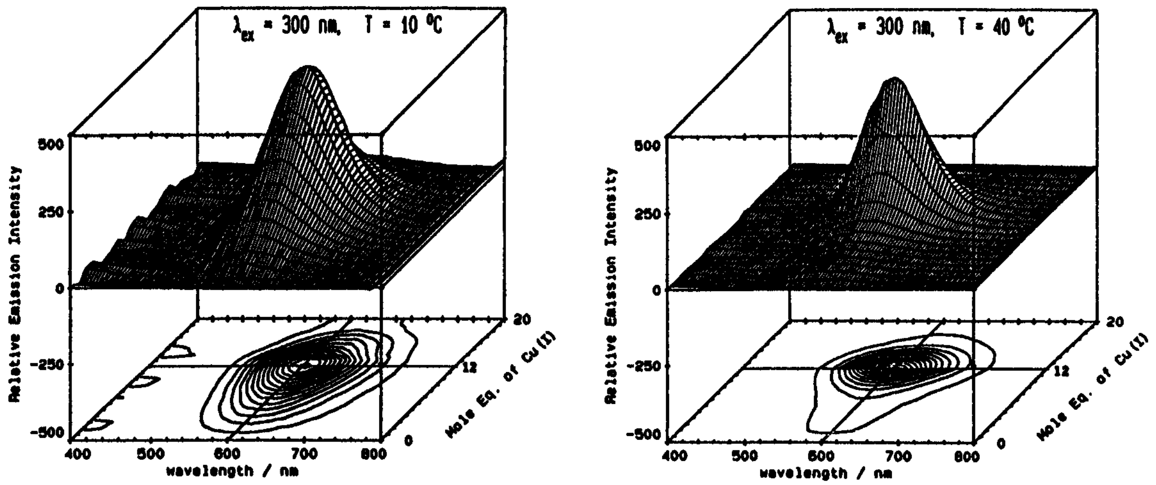

Figure 8. Emission spectral intensity profile recorded as Cu(I) is added to a single solution of rabbit liver $Zn_7$-MT at 10 °C (left) and 40 °C (right). The emission intensity profile is plotted in terms of the quantum yield per Cu(I) added as a function of the Cu:MT molar ratio. The linear increase in quantum yield up to the Cu:MT=12 point observed at 10 °C is replaced by a non-linear dependence on the Cu:MT molar ratio at 40 °C. The emission intensity is rapidly quenched for Cu:MT>12 as the protein unfolds exposing the copper-thiolate cluster structure to the solvent. Reproduced with permission from Green et al., [12b].

of 10 and 40°C and demonstrate that Cu(I) binding to the two domain rabbit liver $Zn_7$-MT takes place in two steps. The key feature is the difference in the intensity in the Cu:MT range of 1-7 at 10 and 40°C. At 40°C the emission per Cu(I) decreases from the 1 Cu(I) to 7 Cu(I) point, whereas, at 10°C there is an almost linear increase in intensity per Cu(I) added up to the 12 Cu(I) point. We have interpreted these effects in terms of the initial and final binding sites for the Cu(I) that is added. Making the assumption (based on analysis of the emission data [12b], that the Cu-S cluster structure in the β domain is about 10 times less emissive than the α domain, we can describe the Cu(I) binding as follows [12b]. At all temperatures, Cu(I) binds statistically to both domains in $Zn_7$-MT at pH 7. At 40°C (and essentially above 10°C) the Cu(I) redistributes to populate preferentially the β domain forming $Zn_4{}^{\alpha}Cu_6{}^{\beta}$-MT; a species that emits much less light than the equivalent species in which the α domain is also populated by Cu(I) which is formed at 10°C.

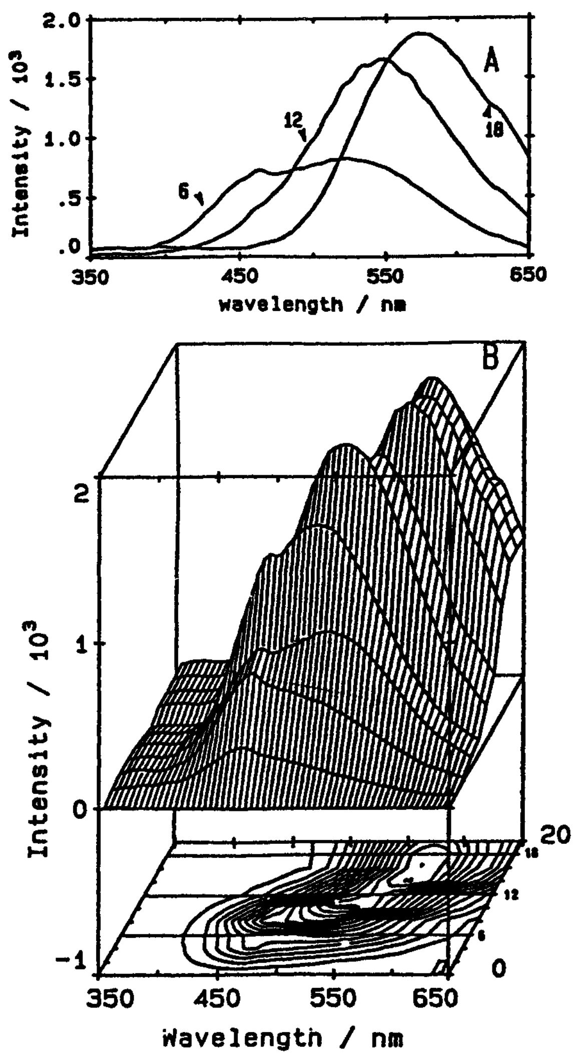

Figure 9. Emission spectral intensity profile recorded as Ag(I) is added to single solutions of rabbit liver apoMT; the spectra were recorded at 77 K. The emission intensity profile is plotted as a function of the Ag:MT molar ratio. The emission intensity rises to a maximum at the Ag:MT=12 point, then falls slightly, accompanied by a red shift in the maximum to the Ag:MT=18point. Reproduced with permission from Zelazowski et al. [18a]

### *Silver and gold binding to metallothionein*

The coordination chemistry of inorganic silver-thiolate complexes is remarkable and unique, in that digonal, trigonal and even tetrahedral geometries are observed [29-32]. Alternating Ag(I)-thiolate chains in inorganic Ag-thiolate complexes are common. It has been found that $[Ag_x(SR)_y]^{(x-y)}$ compounds tend to form aggregates and the degree of aggregation depends upon both the reaction conditions and the nature of the thiolate ligands. In general, thiolate ligands with sterically demanding substituents exhibit a reduced tendency to bridge metal centers, thereby yielding discrete molecular species and reducing the size of molecular $[AgSR]_n$ cycles [30]. The reason that silver-thiolate compounds have a tendency to form aggregates is that there are strong secondary Ag...S interactions which will oppose the formation of discrete molecular cycles, providing a driving force for aggregation and polymerization. However, the steric bulk of the substituent R prevents close approach of the silver units and precludes concomitant Ag...S bridging interactions to form oligomeric aggregates.

The Ag-S bond length in inorganic silver-thiolate clusters is very dependent on the coordination geometry of the silver(I) metal. Generally, the more sulfurs connected to the silver(I), the longer the bond length of Ag-S. However, the correlation between the bond length and coordination number is not straightforward as a result of the sophistication of the coordination environment, such as the secondary Ag...S interactions, the constraints imposed by ring closure, and the effects

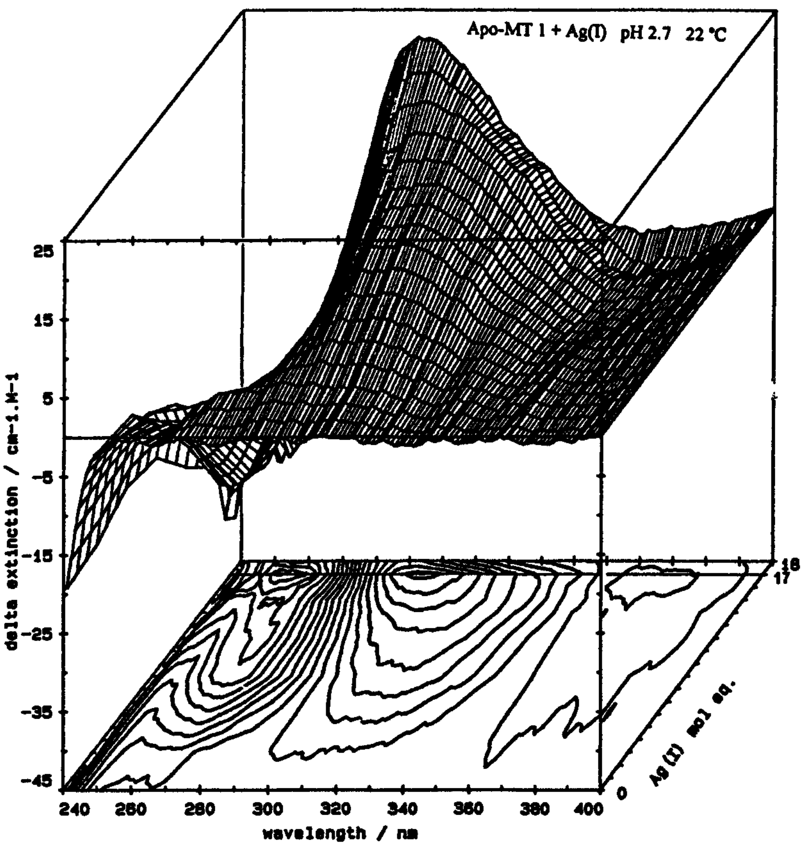

Figure 10. CD spectra recorded as Ag(I) is added to a single solution of rabbit liver apo-MT 1 at 22 °C. The CD intensity profile is plotted as a function of the Ag:MT molar ratio and shows that there is a change in spectral characteristics at the Ag:MT=12 and 17 points. Reproduced with permission from Gui et al. [18c].

of a distorted geometry. Even though average Ag-S distances have been reported as 2.41, 2.53, and 2.60 Å for digonal [30], trigonal [30], and tetrahedral [31] geometries, respectively, individual bond lengths vary sufficiently that they may overlap those of different geometries, Figure 15.

As in inorganic silver-thiolate compounds, digonal and trigonal coordination in silver metallothionein is possible. Circular dichroism and emission spectroscopies have proven to be very effective and precise in identifying structural changes in the metallothionein metal binding sites as metals have been introduced. The metal:protein stoichiometry can be established from the development of CD and emission spectral signals that reach a maximum intensity for a specific Ag:$S_{cys}$ stoichiometry.

Like Cu-MT, mammalian silver metallothioneins emit light, but only at 77 K. Figure 9 shows the emission intensity profile plotted as a function of the Ag(I):MT molar ratio. Spectra were recorded from different solutions formed by adding Ag(I) to apo-MT at room temperature. The emission spectra were recorded from the frozen glassy solutions. Three distinct spectra are observed; at Ag(I):MT of 6, 12, and ca. 18, (see also Figures 10 and 11) Unlike the set of spectra recorded for Cu-MT, Figure 8, the band intensity is not quenched for Ag:MT>12, rather the band maximum red shifts. This spectral change coincides with development of new bands in the CD spectrum measured at room temperature and above, Figures 10 and 11.

The sign and magnitude of the bands in the CD spectrum to the red of 220 nm in metal metallothioneins are dependent on the chirality of the metal binding site as a whole [29]. Therefore, the appearance and disappearance of intensity in individual bands is related to the formation and collapse of the specific metal-thiolate species, respectively.

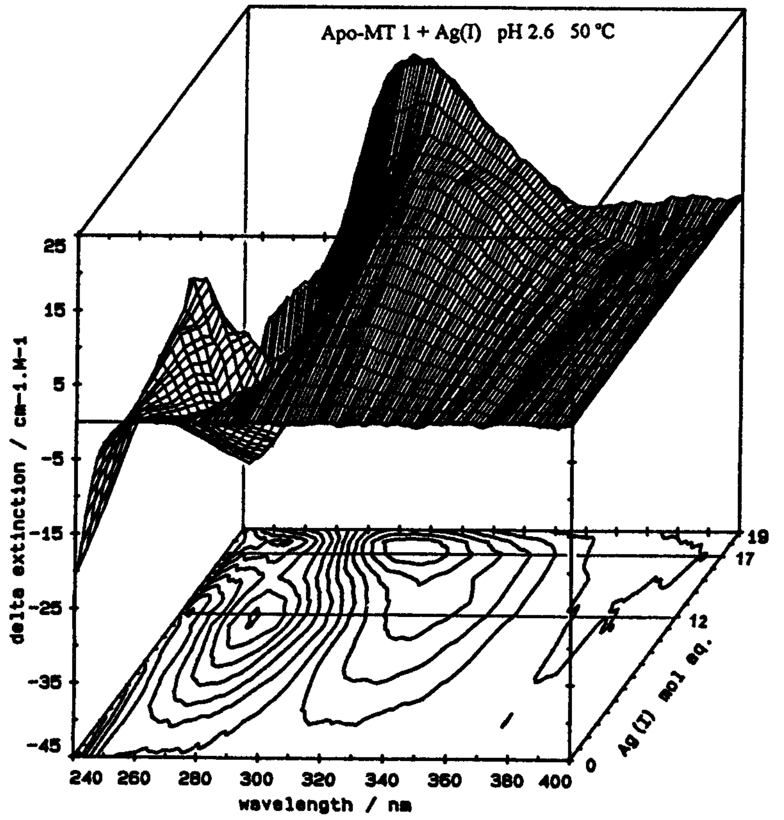

Figure 11. CD spectra recorded as Ag(I) is added to a single solution of rabbit liver apo-MT 1 at 50 °C. The CD intensity profile is plotted as a function of the Ag:MT molar ratio and shows that there is a change in spectral characteristics at the Ag:MT=12 and 17 points. Reproduced with permission from Gui et al., [18c].

Figure 10 shows the three-dimensional CD spectra recorded for a single solution of $Zn_7$-MT 1 at room temperature and pH 2.7. The gradual blue shift of the main CD peak when more than 12 Ag(I) are added to the protein from 310 nm for 12 Ag(I) to 300 nm with 17 Ag(I) identifies the major difference between $Ag_{12}$-MT 1 and $Ag_{17}$-MT 1. The formation of the $Ag_{12}$-MT 1 species, however, is only very poorly resolved by CD spectra recorded at room temperature even though the CD spectrum distinctly indicates the formation of $Ag_{17}$-MT 1. In contrast to the CD spectra shown as Figure 10, the $Ag_{12}$-MT 1 species with its well-defined characteristic CD bands at 315 nm (+, strong) and 265 nm (-,very strong), is unambiguously identified from CD spectra recorded at higher temperatures (50 °C) in Figure 11. As at room temperature, addition of Ag(I) past the Ag:MT=12 point results in formation of $Ag_{17}$-MT 1, with CD band maxima at 370 nm (-, weak) and 300 nm (+, strong). As the pH of the $Zn_7$-MT 1 solutions used in these titrations is quite low, the protein can be regarded as apo-MT 1 because all 7 zinc atoms are released from metallothionein at low pH. We found that the formation of $Ag_{12}$-MT 1 and $Ag_{17}$-MT 1 is better resolved in the CD spectra when Ag(I) is added to apo-MT 1 at low pH values, rather than when Ag(I) is added to $Zn_7$-MT 1 at neutral pH [18b]. This may be due to the fact that the presence of seven zinc atoms in metallothionein interferes in the complete formation of $Ag_{12}$-MT 1 as a result of the competition between the original tetrahedrally-coordinated Zn(II) and the incoming Ag(I). The observation that formation of $Ag_{12}$-MT 1 is more clearly shown in CD spectra when recorded at high temperature than at room temperature may imply that extra energy is required to overcome the relatively high activation energy to form the well-resolved $Ag_{12}$-MT 1 species. The similarity in the Ag-S bond lengths in $Ag_{12}$-MT 1 and in $Ag_{17}$-MT 1, Figure 15 [33] strongly suggest that the Ag(I) adopts a high fraction of digonal geometry in both species.

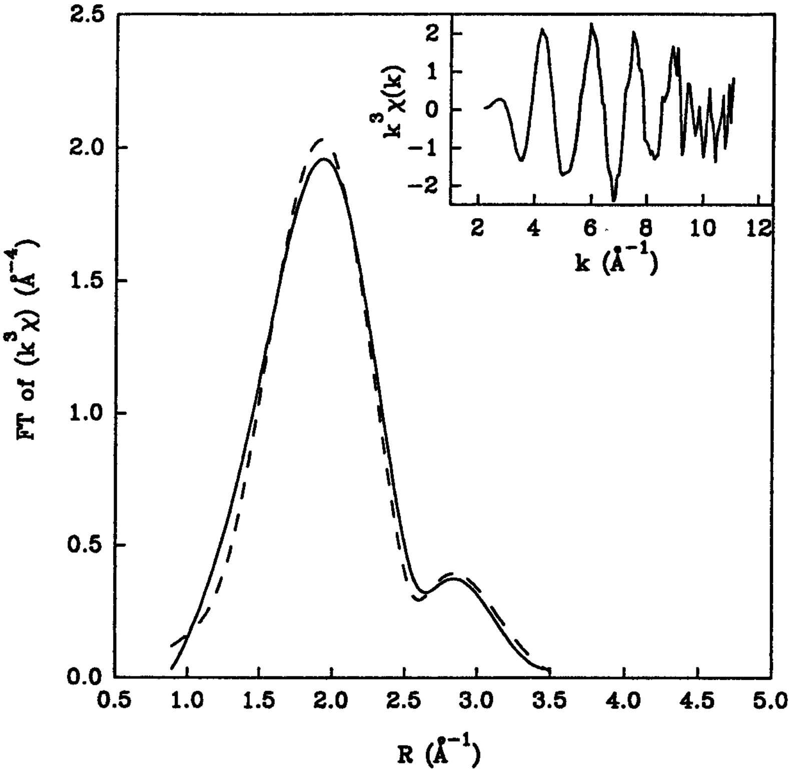

Figure 12. Ag K-edge XAFS raw data in k-space (inset) and the Fourier transform of $k^3\chi(k)$ for $Ag_{17}$-MT at 77 K. Reproduced with permission from Jiang et al. [33].

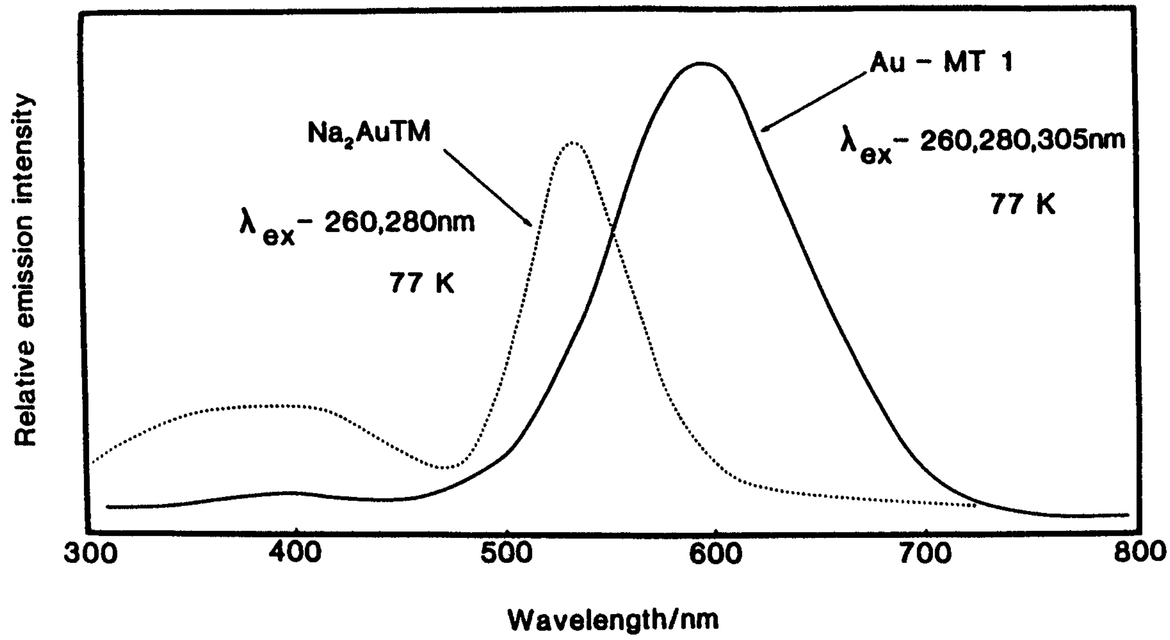

Figure 13. Emission spectra of $Au_7$-MT 1 and $Na_2AuTM$ recorded at 77 K. Reproduced with permission from Stillman et al. [35]

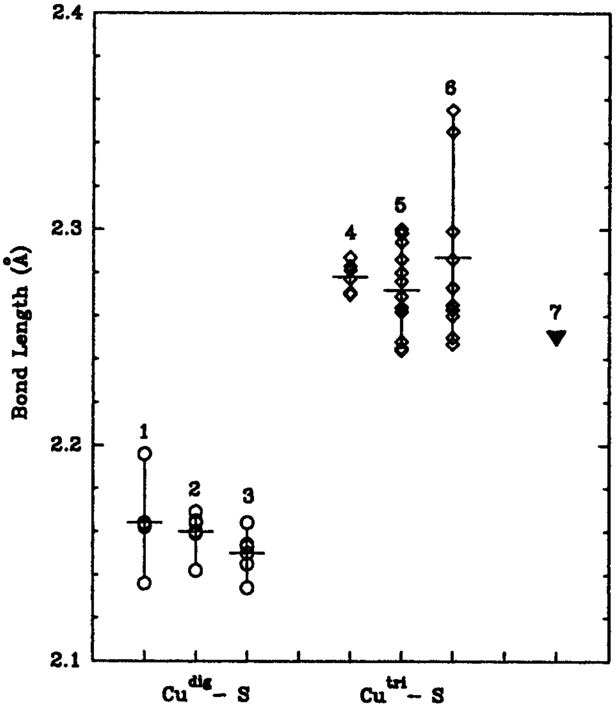

Figure 14. Copper(I)-thiolate bond lengths in six different inorganic model compounds. Data taken from: 1:[42]; 2:[34]; 3: [43 ]; 4: [42]; 5: [44]; 6: [45]; 7: $Cu_{12}$-MT 2 (bond length: 2.25 Å; Ref. 18c)

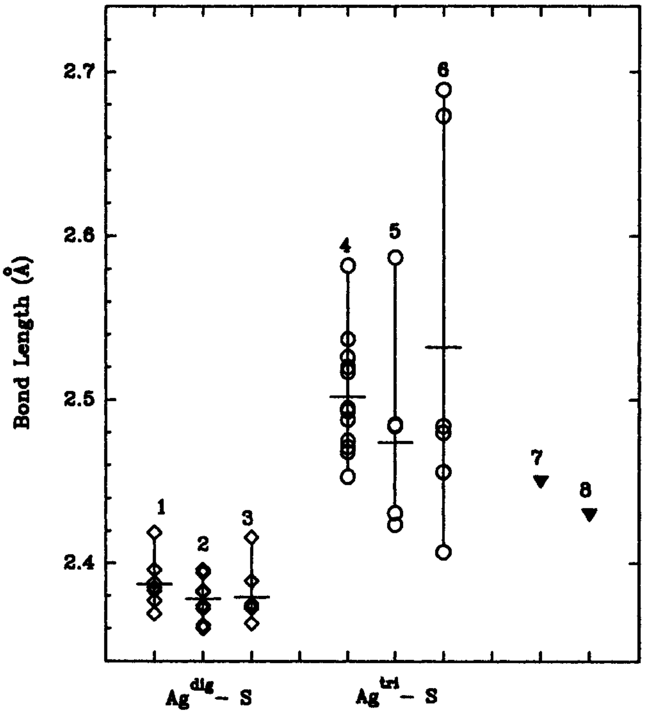

Figure 15. silver(I)-thiolate bond lengths in six different inorganic model compounds. Data taken from: 1:[34]; 2:[30]; 3: [46 ]; 4: [47]; 5: [48]; 6: [49]; 7: $Ag_{12}$-MT 1 (bond length: 2.45 Å; Ref. 33); 8: $Ag_{17}$-MT 1 (bond length: 2.43 Å; Ref. 33)

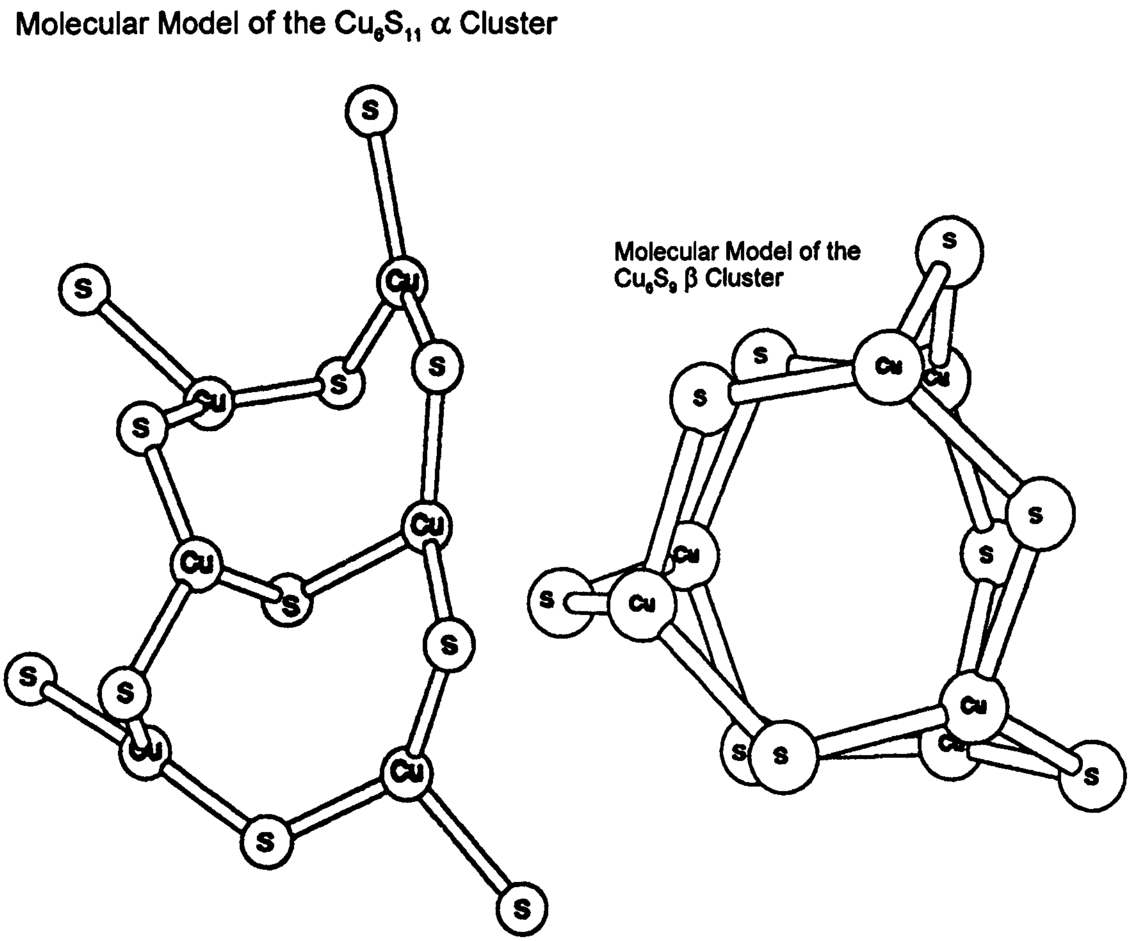

Figure 16. Proposed molecular models of the Cu(I)-thiolate cores based on an energy minimized structure of $Cu_{12}$-MT 2. (A): The $Cu_6S_{11}$ cluster. (B): The $Cu_6S_9$ cluster. Reproduced with permission from Presta et al. [12c].

The local silver-thiolate binding site can be probed by Extended X-ray Absorption Fine Structure (EXAFS) spectroscopy. By measuring Ag K-edge EXAFS, the average bond length of Ag-S can be accurately obtained and the nearest neighbors around Ag(I) can also be estimated. Figure 12 shows the raw data in *k*-space (inset) and the Fourier transform of $k^3\chi(k)$. The Fourier transform spectrum clearly indicates that two separate coordination shells are present. Nonlinear least-squares curve-fitting of the data [33] shows that the nearest neighbor shell in $Ag_{17}$-MT 1 contains two sulfurs at 2.43 ± 0.024 Å with $\sigma^2$ 8.8x10$^{-3}$ Å$^2$ and the second shell contains one silver atom at 2.91 ± 0.05Å with $\sigma^2$ 13.7x10$^{-3}$ Å$^2$. The bond length of the Ag-S shell falls in the range of characteristic values for digonal coordination [30] and it is consistent with the coordination number of 2 obtained from the EXAFS analysis. We recently also measured the Ag K-edge EXAFS of $Ag_{12}$-MT 1 and a preliminary analysis of the EXAFS data (not shown) shows that Ag(I) in $Ag_{12}$-MT 1 surprisingly has the similar local environment to Ag(I) in $Ag_{17}$-MT 1. The bond lengths of Ag-S and Ag...Ag for $Ag_{12}$-MT 1 are 2.45 Å and 2.91 Å, respectively. At first glance, these results seem to be contrary to the results revealed from the CD spectra. As mentioned above, CD spectroscopy provides information about the protein's three-dimensional structure as a whole, while EXAFS spectroscopy probes the local structure of the metal binding site of the protein. Hence, these two techniques give complementary structural information on metallothionein. As displayed by the CD spectra, the wrapping structure in $Ag_{12}$-MT 1 is different from that of $Ag_{17}$-MT 1. However, due to the complexity of the silver-thiolate coordination phenomenon as illustrated by inorganic silver-thiolate compounds, it is not surprising that the average Ag-S bond length in the local binding site of $Ag_{12}$-MT 1 from EXAFS measurements does not change when compared to that of $Ag_{17}$-MT 1. Another possibility is that Ag(I) in $Ag_{12}$-MT 1 may have a T-shaped geometry [34], in which one of three Ag-S bonds is so long (about 3 Å) that it is hardly detected by EXAFS spectroscopy.

Finally, Figure 13 shows the emission spectra recorded for glassy solutions of Au-MT and gold thiomalate at 77 K [35]. As with Cu-MT and Ag-MT, the band maximum exhibits a 300 nm Stoke's shift: excitation at 300 nm results in emission at 600 nm.

### *Summary of structural properties*

The Cu-S bond length range in copper(I)-thiolate inorganic compounds with digonal and trigonal coordination geometries is shown in Figure 14. It is obvious that the average Cu-S bond length for digonal geometry is about 0.1 Å shorter than that found in trigonal geometry. Furthermore, the variation of Cu-S bond lengths in trigonal coordination is much larger than that found for the digonal geometries. This may be ascribed to the fact that the complexes based on trigonal Cu(I)-thiolate coordination have greater distortions due to the intramolecular repulsions between

Table 1. Metal-thiolate coordination geometries reported for metals bound to mammalian and yeast metallothioneins.

| | ***tetrahedral*** | ***trigonal*** | ***digonal*** | ***mixed*** |
|---|---|---|---|---|
| Cd(II) in mammalian liver protein | $Cd_7$-MT [5-7] | | | |
| Co(II) in mammalian liver protein | $Co_7$-MT [38] | | | |
| Zn(II) in mammalian liver protein | $Zn_7$-MT [6c,7] | | | |
| Hg(II) in mammalian protein | $Hg_7$-MT [16a]<br>EXAFS data suggest that the structure is distorted from tetrahedral geometry [17] | $Hg_{11}$-MT [16a] | $Hg_{18}$-MT [16b,17] | |
| Ag(I) in mammalian liver protein | | | $Ag_{17}$-MT [18c,33] | $Ag_{12}$-MT [18c,33] |
| Cu(I) in mammalian liver protein | | $Cu_{12}$-MT [12c,15] | | $Cu_{15}$-MT [12c]<br>Cu-MT [39] |
| Au(I) in horse kidney protein | | | Au,Zn,Cd-MT (Au-S=229 pm) [32]<br>$(TmSAu)_x$MT (Au-S=230 pm [32] | |
| Cu(I) in yeast protein | | $Cu_8$-MT [40] | | $Cu_7$-MT [41] |
| Ag(I) in yeast protein | | | | $Ag_7$-MT [41] |

thiolate substituents. The Cu-S bond length in $Cu_{12}$-MT 2 (compound 7) was determined by sulfur K-edge EXAFS measurements to be 2.25 Å [18c]. Evidently this value falls in the range of bond lengths characteristic of trigonal Cu(I)-thiolate geometries.

The analogous data for silver(I)-thiolate compounds are presented in Figure 15. Similar to the data for the Cu(I)-thiolate compounds, the average Ag(I)-S bond lengths for digonal coordination are approximately 0.1 Å shorter than those for trigonal geometries. The very large variation in Ag(I)-S bond lengths for the compounds with trigonal coordination may result from strong secondary Ag...S interactions, and the intramolecular repulsions between thiolate substituents. The Ag(I)-S bond lengths for $Ag_{12}$-MT 1 and $Ag_{17}$-MT1 determined from Ag K-edge EXAFS measurements are 2.45 Å and 2.43 Å, respectively [33].

Modeling the Cu(I)-thiolate structures of $Cu_{12}$-MT based on synthetic complexes may prove helpful in understanding the binding and functional properties of both copper and silver metallothioneins. Dance has noted several recurring structural motifs in synthetic Cu(I)-thiolate complexes [36] that include: (a) $(\mu\text{-SR})_3(CuSR)_3$ rings and (b) $(\mu\text{-SR})_4Cu_4$ rings. These structures display trigonal coordination for the Cu(I) atoms, the latter motif often including terminal ligation of the Cu(I) by other types of ligands, such as phosphines. Table 1 lists proposed coordination geometries of a number of metallothioneins. Trigonal coordination predominates with the Cu(I) and Ag(I) proteins.

An energy-minimized model of the Cu(I)-thiolate clusters in rabbit liver $Cu_{12}$-MT 2a, described previously by Presta et al. [12c], is shown in Figure 16. The $Cu_6S_{11}$ cluster (Figure 16a) representing the $\alpha$ domain cluster, is composed of two $Cu_4S_{cys4}$ rings, connected by a bridging S. There are also four terminal cysteine thiolate ligands which bind the remaining Cu(I) atoms, so that all copper atoms are coordinated to three thiolates. The average Cu-bridging S bond length in this structure is 220(1) pm, while the average bond length between the Cu(I) atoms and terminal sulfurs is 251(1) pm. Therefore, this structure contains the $(\mu\text{-SR})_4Cu_4$ ring motif common to several synthetic Cu(I)-thiolate complexes.

The $Cu_6S_9$ cluster (Figure 16b) representing the $\beta$ domain cluster, is a cage displaying two six-membered rings with alternating Cu and S atoms, forming front and back "faces" of the cage. These rings or faces are interconnected by bridging sulfur atoms between the opposite Cu atoms, such that three $Cu_4Scys_4$ rings make up the sides of the cage and all Cu(I) atoms have trigonal geometry. The average Cu-S bond length in this cluster is 219(2) pm. This structure, which is a distorted prism of copper(I) ions, has only bridging thiolate ligation, in contrast to the proposed $\alpha$ domain cluster which has four terminal thiolates, and displays both the $(\mu\text{-SR})_4Cu_4$ ring and the $(\mu\text{-SR})_3(CuSR)_3$ ring motifs that are common among synthetic Cu(I)-thiolate complexes.

This model of the protein may be used to account for several interesting properties of copper-metallothioneins. First, although the metallothionein peptide chain is rather short, it is quite efficient at embedding the Cu(I)-thiolate clusters, in large part preventing solvent access to these cores. For this reason, $Cu_{12}$-MT luminesces brightly at about 600 nm ($\lambda_{excitation}$ = 300 nm) even in solution [12b], Figure 8. Synthetic Cu(I) complexes normally luminesce only in the frozen glassy state [37].

Within this general encapsulation of the Cu(I)-thiolate cores by the peptide chain, there do exist small openings in each domain where some sulfur and copper atoms are visible, and presumably accessible. The fact that the $Cu_6Scys_{11}$ $\alpha$ domain cluster has four terminal thiolates which can coordinate another metal suggests that these exposed sulfur atoms in the $\alpha$ domain may be the most susceptible to bind extra metal ions in forming the $Cu_{15}$-MT and $(Cu_6Cd_4)^{\alpha}(Cu_6)^{\beta}$-MT species [12c]. The exposed Cu(I) atoms may also represent the sites of interactions with small ligands like glutathione (Presta and Stillman, unpublished results), which would be able to penetrate the opening, and thus could be involved in metal-exchange reactions [50].

### *Acknowledgments*

We gratefully acknowledge financial support from NSERC of Canada for operating funds (to M.J.S.), a graduate scholarship (to P.A.P.) and a postdoctoral fellowship (to D-T.J), the Province of Ontario for a fee bursary (to Z.G.), and the Academic Development Fund at the UWO for an equipment grant to M.J.S.. We also wish to thank Drs. G.M. Bancroft, M. Kasrai, and T.K. Sham for collaboration with the XAS experiments. The EXAFS data were recorded at the Brookhaven National laboratory in collaboration with Dr. S.M. Heald. We thank Anna Rae Green for permission to reproduce Figure 8.

### *References*

1. Margoshes, M. and Vallee, B.L., J. Am. Chem. Soc., 79 (1957) 4813-4814.
2. Kagi, J.H.R. and Vallee, B.L., J. Biol. Chem., 235 (1960) 3460-3465.
3. Kagi, J.H.R. and Vallee, B.L., J. Biol. Chem., 236 (1961) 2435-2442.
4. a) Metallothionein, Kagi, J.H.R. and Nordberg, M., eds., Birkhauser Verlag, Basel (1979). b) Biological Roles of Metallothionein, Foulkes, E.C., Ed. Elsevier, Amsterdam (1982). c) Metallothionein II, Kagi, J.H.R. and Kojima, Y., eds. Birkhauser Verlag, Basel (1987). d) Metallobiochemistry Part B. Metallothionein and Related Molecules. Methods in Enzymology, vol. 205, Riordan, J.F. and Vallee, B.L., eds., Academic Press, New York (1991). e) Metallothioneins, Stillman, M.J., Shaw, C.F., and Suzuki, K.T., eds. VCH Publishers, New York (1992). f) Metallothionein III, Suzuki, K.T., Imura, N. and Kimura, M., eds., Birkhauser Verlag, Basel (1993).
5. Otvos, J.D. and Armitage, I.M., Proc. Natl. Acad. Sci. USA, 77 (1980) 7094-7098.
6. a) Frey, M.H., Wagner, G., Vasak, M., Sorensen, O.W., Neuhaus, D., Worgotter, E., Kagi, J.H.R., Ernst, R.R., and Wuthrich, K., J. Am. Chem. Soc., 107 (1985) 6847-6851. b) Arseniev, A., Schultze, P., Worgotter, E., Braun, E., Wagner, G., Vasak, M., Kagi, J.H.R., and Wuthrich, K., J. Mol. Biol., 201 (1988) 637-657). c) Messerle, B.A., Schaffer, A., Vasak, M., Kagi, J.H.R., and Wuthrich, K., J. Mol. Biol., 225 (1992) 433-443.
7. a) Robbins, A.H., McRee, D.E., Williamson, M., Collett, S.A., Xuong, N.H., Furey, W.F., Wang, B.C., and Stout, C.D., J. Mol. Biol., 221 (1991) 1269-1293. b) Robbins, A.H. and Stout, C.D., In M.J. Stillman, C.F. Shaw III, and K.T. Suzuki (Eds.), Metallothioneins, VCH Publishers, New York, 1992, Chapter 3, pp. 31-54.
8. Kagi, J.H.R., In K.T. Suzuki, N. Imura, and M. Kimura (Eds.), Metallothionein III, Birkhauser Verlag, Basel, 1993, pp 29-55.
9. Kagi, J.H.R. and Kojima, Y., Experientia Suppl., 52 (1987) 25-61.
10. Stillman, M.J., Cai, W., and Zelazowski, A.J., J. Biol. Chem., 262 (1987) 4538-4548.
11. Nielson, K.B. and Winge, D.R., J. Biol. Chem., 259 (1984) 4941-4946.
12. a) Stillman, M.J. and Zelazowski, A.J. J. Biol. Chem. 263 (1988) 6128-6133. b) Green, A.R., Presta, A., Gasyna, Z., and Stillman, M.J., Inorg. Chem., 33 (1994)4159-4168. c) Presta, A., Green, A.R., Zelazowski, A., and Stillman, M.J. submitted for publication.
13. Vasak, M., Overnell, J., and Good, M., Experientia Suppl., 52 (1987) 179-189.
14. a) Nielson, K.B., Atkin, C.L., and Winge, D.R., J. Biol. Chem., 260 (1985) 5342-5350. b) Li, Y.-J. and Weser, U., Inorg. Chem., 31 (1992) 5526-5533.
15. a) George, G.N., Winge, D.R., Stout, C.D., and Cramer, S.P., J. Inorg. Biochem., 27 (1986) 213-220. b) Abrahams, I.L., Bremner, I., Diakun, G.P., Garner, C.D., Hasnain, S.S., Ross, I., and Vasak, M., Biochem. J., 236 (1986) 585-589.
16. a) Cai, W. and Stillman, M.J., J. Am. Chem. Soc., 110 (1988) 7872-7873. b) Lu, W., Zelazowski, A.J., and Stillman, M.J., Inorg. Chem., 32 (1993) 919-926.
17. Jiang, D.T., Heald, S.M., Sham, T.K., and Stillman, M.J., J. Am. Chem. Soc. 116 (1994) 000.
18. a) Zelazowski, A.J., Gasyna, Z., and Stillman, M.J., J. Biol. Chem., 264 (1989) 17091-17099. b) A.J. Zelazowski and M.J. Stillman, Inorg. Chem., 31 (1992) 3363-3370. c) Gui, Z., Kasrai, M., Green, A. R., Yang, B.X., Feng, X.H., Bancroft, G.M., and Stillman, M.J., submitted for publication.

19. a) Gasyna, Z., Zelazowski, A., Green, A.R., Ough, E.A., and Stillman, M.J., Inorg. Chim. Acta, 153 (1988) 115-118. b) Stillman, M.J. and Gasyna, Z., Meth. Enzymol., 205 (1991) 540-555.
20. Stillman, M.J., Gasyna, Z., and Zelazowski, A.J., FEBS Lett., 257 (1989) 283-286.
21. Zelazowski, A.J., Szymanska, J.A., and Witas, H., Prep. Biochem., 10 (1980) 495-505.
22. Ellman, G.L., Arch. Biochem. Biophys., 82 (1959) 70-77.
23. Birchmeier, W. and Christen, P., FEBS Lett., 18 (1971) 209-213.
24. Hemmerich, P. and Sigwart, C., Experientia, 19 (1963) 488-489.
25. Gasyna, Z., Browett, W.R., Nyokong, T., Kitchenham, B., and Stillman, M.J., Chemom. Intell. Lab. Syst., 5 (1989) 233-246.
26. Lu, W., Kasrai, M., Bancroft, G.M., Stillman, M.J., and Tan, K.H., Inorg. Chem., 29 (1990) 2561-2563.
27. Lu, W. and Stillman, M.J., J. Am. Chem. Soc., 115 (1993) 3291-3299.
28. a) Beltramini, M. and Lerch, K., Biochem., 22 (1983) 2043-2048. b) Beltramini, M., Giacometti, G.M., Salvato, B., Giacometti, G., Munger, K., and Lerch, K., Biochem. J., 260 (1989) 189-193.
29. Stillman, M.J., In M.J. Stillman, C.F. Shaw III, and K.T. Suzuki (Eds.), Metallothioneins, VCH Publishers, New York, 1992, Chapter 4, pp. 55-127.
30. Tang, K., Aslam, M., Block, E., Nicholson, T., and Zubieta, J., Inorg. Chem., 26 (1987) 1488-1497.
31. Schuerman, J.A., Fronczek, F.R., and Selbin, J., Inorg. Chim. Acta, 160 (1989) 43-52.
32. Laib, J.E., Shaw, C.F. III, Petering, D.H, Eidness, M.K., Elder, R.C., and Garvey, J.S., 24 (1985) 1977-1986).
33. Jiang, T.D., Gui, Z.Q., Heald, S.M., Sham, T.K., and Stillman, M.J., The 8th International Conference on XAFS, Berlin, Germany (1994).
34. Block, E., Gernon, M., Kang, H., Ofori-Okai, G., and Zubieta, J. Inorg. Chem. 28 (1989) 1263-1271.
35. Stillman, M.J., Zelazowski, A.J., Szymanska, J., and Gasyna, Z., Inorg. Chim. Acta, 161 (1989) 275-279
36. Dance, I., Fisher, K., and Lee, G. (1992) in Metallothioneins (Stillman, M.J., Shaw, C.F., III, and Suzuki, K.T., Eds.) pp. 284-345, VCH Publ.: New York.
37. a) Buckner, M.T. and McMillin, D.R., J. Chem. Soc., Chem. Comm., (1978) 759-761. b) Henary, M. and Zink, J.I., J. Am. Chem. Soc., 111 (1989) 7407-7411. c) Sabin, F., Ryu, C.K., Ford, P.C., and Vogler, A. Inorg. Chem., 31 (1992) 1941-1945.
38. Bertini, I., Luchinat, C., Messori, L., and Vasak, M. Eur. J. Biochem., 211 (1993) 235-240.
39. Pickering, I.J., George, G.N., Dameron, C.T., Kurz, B., Winge, D.R., and Dance, I.G., J. Am. Chem. Soc., 115 (1993) 9498-9505.
40. Winge, D.R., Meth. Enzymol., 205 (1991) 458-469.
41. Narula, S.S., Winge, D.R., and Armitage, I.M. Biochem., 32 (1993) 6773 -6787.
42. Bowmaker, G.A., Clark, G.R., Seadon, J.K., and Dance, I.G., Polyhedron, 3 (1984) 535-544.
43. Block, E., Kang, H., Ofori-Okai, G., and Zubieta, J., Inorg. Chim. Acta 167 (1990) 147-148.
44. Nicholson, J.R., Abrahams, I.L., Clegg, W., and Garner, C.D., Inorg. Chem., 24 (1985) 1092-1096.
45. Dance, I.G., Bowmaker, G.A., Clark, G.R., and Seadon, J.K., Polyhedron, 2 (1983) 1031-1043.

46. Dance, I.G., Fitzpatrick, L.J., Craig, D.C., and Scudder, M.L., Inorg. Chem., 28 (1989) 1853-1861.
47. Dance, I.G., Aust. J. Chem., 31 (1978) 2195-2206.
48. Dance, I.G., Inorg. Chem., 20 (1981) 1487-1492.
49. Dance, I.G., Fitzpatrick, L.J., and Scudder, M.L., Inorg. Chem., 23 (1984) 2276-2281
50. a) Brouwer, M. and Brouwer-Hoexum, T., Arch. Biochem. Biophys., 290 (1991) 207-213. b) Brouwer, M. and Brouwer-Hoexum, T., Biochemistry, 31 (1992) 4096-4102..

